# A modular platform for nucleic acid-driven multimerization of nanobodies for advanced molecular imaging

**DOI:** 10.1016/j.omtn.2024.102367

**Published:** 2024-11-06

**Authors:** Laura P. Rebolledo, Nathalia Leal Santos, Kirill A. Afonin

**Affiliations:** 1 Nanoscale Science Program, Department of Chemistry, University of North Carolina at Charlotte, Charlotte, NC 28223, USA; 2 Center for Translational Research in Oncology (LIM24), Instituto do Câncer do Estado de São Paulo, Hospital das Clínicas da Faculdade de Medicina da Universidade de São Paulo, Comprehensive Center for Precision Oncology, Universidade de São Paulo, São Paulo, Brazil

## Main text

Affordable and robust molecular imaging plays a critical role in cancer detection and treatment selection, offering precise visualization of tumor biology and behavior. Further advancements in this field can lead to more accurate diagnoses, better assessments of cancer stage and aggressiveness, improved treatment planning, and personalized therapy for better patient outcomes.

Current techniques rely on targeting ligands, molecules that bind specifically to receptors overexpressed in cancer cells. Monoclonal antibodies are widely used for this purpose due to their high affinity for cancer biomarkers. Still, their relatively large size leads to poor tissue penetration and uneven tumor accumulation.^1^ These limitations result in delayed imaging with less effective treatment management.

**Figure 1:**
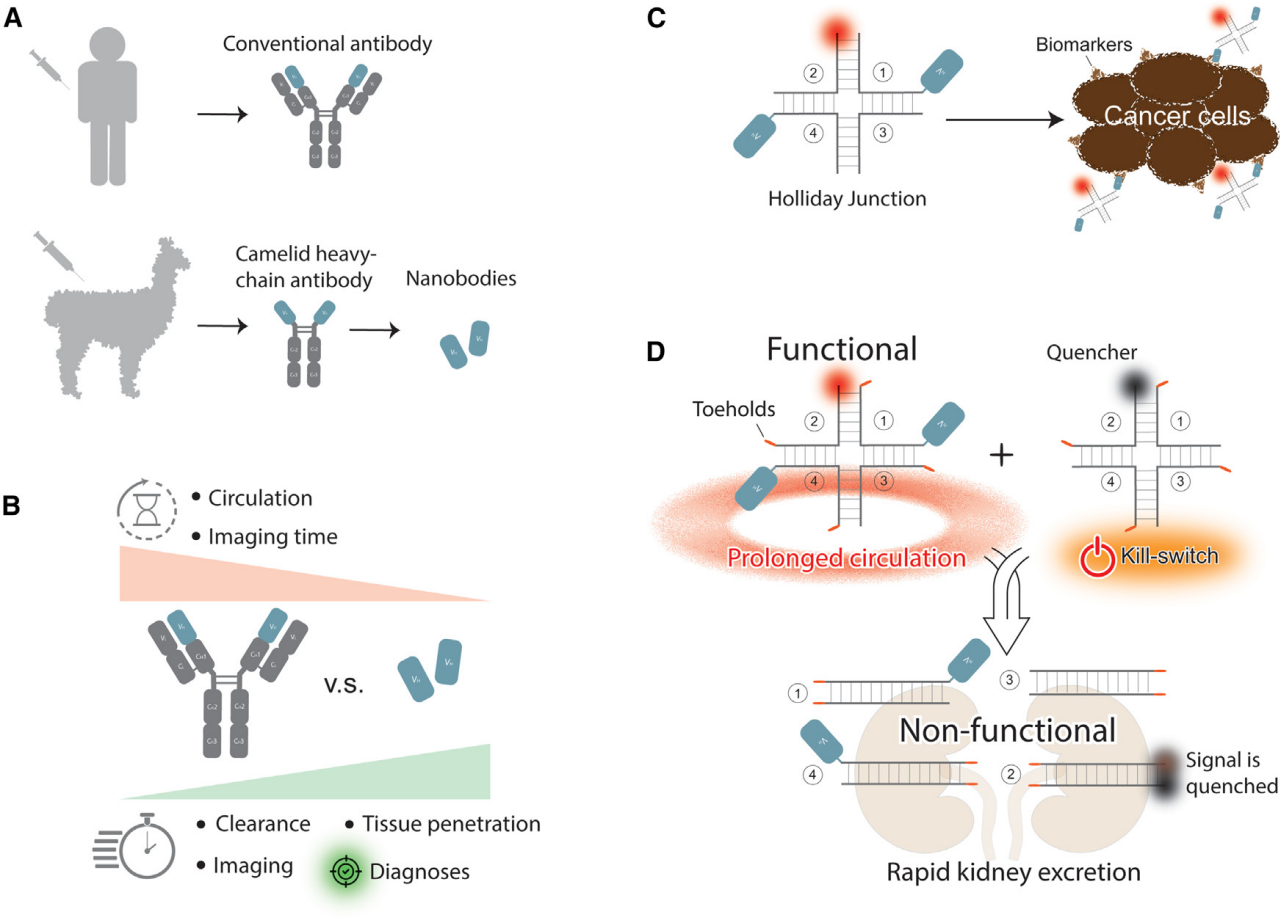
Modular platform with nucleic acid-driven multimerization of nanobodies for advanced molecular imaging (A) Schematic representation of the human antibody and camelid nanobody and (B) comparison of their properties for molecular imaging. (C) Schematics of the investigated DNA nanoassemblies displaying biparatopic configuration of nanobodies and (D) proposed implementation of kill switches causing inactivation of circulating constructs and producing smaller nonfunctional assemblies for accelerated renal excretion.

In the September issue of *Molecular Therapy Nucleic Acids*, Teodori et al. offer a promising innovation in this area using nanobodies, small antibody fragments derived from camelids (Figure 1A). Due to their reduced size, nanobodies retain the specificity of full-sized antibodies but offer advantages in higher tissue penetration while providing much faster imaging. These attributes make nanobodies particularly well suited for molecular imaging applications, where rapid and precise targeting of tumor cells is essential (Figure 1B).^2^^,^^3^

However, since the rapid circulation in the bloodstream can limit nanobody accumulation at specific sites, thus reducing imaging contrast, multimerization strategies are being explored to enhance nanobody binding through avidity while tuning imaging agents’ circulation time and valency. A research team led by Jørgen Kjems has developed a novel technology (Figure 1C) that uses a Holliday junction as a nucleic acid-based scaffold to guide the multimerization of structures composed of fluorescent tags and nanobodies: either homobivalent (with two nanobodies targeting the same human epidermal growth factor receptor 2 [HER2] epitope) or biparatopic (targeting different HER2 epitopes). HER2 is a key biomarker overexpressed in certain aggressive cancers, including breast cancer, making it a crucial target for both imaging and therapy. The findings from this comprehensive experimental study reveal that biparatopic constructs show enhanced binding strength to HER2, surpassing the sensitivity of traditional mAbs.

### Clinical and theragnostic potential of the developed platform

Nanobodies hold potential not only for diagnostic applications but also as theranostic agents. Theranostics can combine diagnosis and therapy in a single formulation, offering both targeted treatment and real-time tracking of therapeutic efficacy. The biparatopic constructs from the study by Teodori et al. could be conjugated with one or more antitumor agents to deliver a targeted therapeutic payload while simultaneously allowing for high-resolution imaging of tumor response. Due to the programmability of all used components, this approach could extend beyond HER2^+^ breast cancer, targeting other biomarkers across various cancer types, thus broadening the scope of precision oncology.

To address the challenges posed by the larger size of the multimerized biparatopic constructs, one potential strategy inspired by other studies could be using a “kill switch” mechanism.^4^ This programmable platform would take a larger structure and conditionally break it down into smaller, more manageable components, improving their overall clinical utility (Figure 1D). The kill switch mechanism functions via toehold-mediated isothermal strand displacement, where complementary sequences bind to single-stranded DNA regions within the larger functional construct, triggering its controlled disassembly into smaller non-functional DNA duplexes. The implementation of this approach would allow for regulated and more rapid renal clearance, thus offering significant advantages for both diagnostic and therapeutic applications.

In a clinical context, the reported approach is particularly valuable for detecting microscopic tumor lesions and spatial heterogeneity in biomarker expression across tumor cores and peripheries. This could significantly reduce misclassification in cancer diagnosis.^5^ The faster clearance of the smaller inactive structures, aided by the kill switch, would further reduce radiation exposure, making the platform safer for patients. Additionally, the programmability of the kill switch could improve patient outcomes by optimizing nanobody-based constructs for various tumor types, increasing their adaptability to different clinical scenarios.

Looking forward, this “plug-and-play” nanobody platform has the potential to transform molecular imaging and cancer diagnostics. With further optimization, it could offer a highly versatile tool for detecting cancer at earlier stages, improving treatment outcomes and integrating diagnosis with targeted therapy. As research in this area progresses, it is likely that these advances will move beyond HER2 and into broader applications, providing a more comprehensive toolkit for precision medicine.

## Acknowledgments

K.A.A. and L.P.R. thank the National Institute of General Medical Sciences of the National Institutes of Health under Award Number R35GM139587 for their support. N.L.S. was supported by Fundação de Amparo a Pesquisa do Estado de São Paulo, process number 2024/09411-8. The content of this publication does not necessarily reflect the views or policies of the US Department of Health and Human Services, nor does mention of trade names, commercial products, or organizations imply endorsement by the US government.

## Declaration of interests

The authors declare no competing interests.
